# Scrutinizing the impact of two self-regulation policies on unhealthy food marketing in children’s popular television in Malaysia: a multiple-year repeated evaluation using a harmonized protocol

**DOI:** 10.1080/16549716.2025.2543617

**Published:** 2025-08-21

**Authors:** Gild Rick Ong, Mohd Jamil Sameeha, Sreelakshmi Sankara Narayanan, Karuthan Chinna, Bridget Kelly, Sally Mackay, Boyd Swinburn, Tilakavati Karupaiah

**Affiliations:** a School of Biosciences, Faculty of Health and Medical Sciences, Taylor’s University, Subang Jaya, Malaysia; b Nutritional Sciences Programme, Centre for Community Health Studies (ReaCH), Faculty of Health Sciences, Universiti Kebangsaan Malaysia, Kuala Lumpur, Malaysia; c Food Security and Nutrition Impact Lab, Taylor’s University, Subang Jaya, Malaysia; d Faculty of Business and Management, UCSI University, Kuala Lumpur, Malaysia; e Faculty of the Arts, Social Sciences and Humanities, Early Start, School of Health and Society, University of Wollongong, Wollongong, NSW, Australia; f School of Population Health, The University of Auckland, Auckland, New Zealand

**Keywords:** Asia, nutrient profile model, fast food, peak viewing time, persuasive marketing

## Abstract

**Background:**

Regulating unhealthy food marketing is critical as it is a recognized driver of childhood obesity. Two voluntary self-regulatory policies governing food advertising in the media were introduced in Malaysia in 2008 and 2013.

**Objectives:**

To assess food advertising on Malaysian children’s popular television channels across a decade using the standardized INFORMAS protocol.

**Methods:**

The main dataset was collected cross-sectionally from 2020 to 2022 evaluating three television channels. Additionally, a retrospective comparison between the 2022 and 2012 datasets was limited to two channels commonly available for both years. Advertised foods were classified as permitted (healthy) or not-permitted (unhealthy) using a nutrient profile model of the World Health Organization. We compared advertising rates and use of persuasive marketing techniques during children’s peak viewing time (PVT) versus non-PVT.

**Results:**

Unhealthy food advertising rates remained significantly higher than healthy food for all years of measurement (all *p* < 0.001). For the main dataset years (2020, 2021, 2022), unhealthy food advertising rates were 84%, 65%, and 72% higher during PVT compared to non-PVT (all *p* < 0.001). For all 3 years, the use of persuasive marketing techniques engaged in unhealthy food advertising during PVT was greater compared to non-PVT (all *p* < 0.05), whereas this pattern was not observed for the 2012 dataset (all *p* > 0.05). In 2022, fast foods emerged as the most frequently advertised unhealthy food (1.33 ± 2.23 ads/h/channel), a six-fold increase compared to 2012 (0.21 ± 0.47 ads/h/channel).

**Conclusions:**

Unhealthy food advertising dominates Malaysian children’s popular television channels, especially during PVT despite the presence of voluntary self-regulatory policies. These findings underscore the need for government-led mandatory regulations to control unhealthy food marketing targeting children.

## Paper Context

**Main findings:** Unhealthy foods dominated television food advertising on Malaysian children’s popular television channels compared to healthy foods over a decade since the introduction of two voluntary self-regulatory policies, with higher rates broadcasted during children’s peak viewing time and unhealthy fast-food advertising increasing six-fold in 2022 compared to 2012.

**Added knowledge:** This study exposed the inadequacy of the two self-regulatory policies in protecting children from the unhealthy television food advertising over the span of a decade using a robust and standardized monitoring protocol.

**Global health impact for policy and action:** The evidence from this study will be strategized for advocating mandatory policies to government to protect children from the unhealthy food advertising, in line with the recommendations of the World Health Organization.

## Background

In Malaysia, data from the National Health and Morbidity Survey showed that rates of childhood obesity have doubled among children aged 5–17 years within a decade, increasing from 6.1% in 2011 to 14.8% in 2019 [1]. Children who are overweight or obese are at greater risk of developing non-communicable diseases (NCDs) in adulthood [2,3], including type 2 Diabetes mellitus [4,5], hypertension [6], dyslipidemia [7,8] and metabolic dysfunction – associated fatty liver disease [9].

The ‘obesogenic environment’ has been recognized as a key driver of the growing rates of childhood obesity [10–12]. Food marketing is a key influencer of the food environment, communicating information and ideas about foods and brands that are available and desirable [13]. Exposure to marketing influences children’s food preferences, food purchases and requests, food choices, and ultimately increasing caloric consumption from unhealthy foods [14–16]. Television remains a major source of advertising expenditure globally, despite the rise in digital media marketing [17]. Earlier studies have indicated that unhealthy foods constitute the majority of television food advertising in many countries [18,19], including Malaysia [20–22]. These unhealthy foods are high in total and saturated fats, sugar, and salt, and include items such as confectionery, savory snacks, and sugar-sweetened beverages. Restricting the marketing of unhealthy foods to children is recognized as a critical strategy for preventing childhood obesity [13].

Recognizing the harmful impact of unhealthy food marketing on children, international bodies have called on governments to impose regulations to restrict the marketing of unhealthy foods to children [13]. Significant policy progress toward imposing mandatory regulations on television food advertising has been observed in Western countries. For example, Chile banned the advertising of unhealthy foods on television from 6 am to 10 pm [23] and the United Kingdom introduced legislation to ban junk food advertising on television before 9 pm, set to come into force from October 2025 [24]. However, regulations on unhealthy food marketing in Asia remain weak, with most countries either having no policy or rely on industry self-regulation [25,26]. The latter scenario is true for Malaysia.

The Malaysian government first articulated concerns about fast-food advertising on broadcast media in the National Plan of Action for Nutrition of Malaysia (NPANM) 1996–2000 [27] and further outlined activities to support regulation of food marketing on mass media in the second NPANM 2006–2015 [28]. This led to the Malaysian government introducing the ‘Guideline on the Advertising and Nutrition Information Labelling of Fast Foods’ (henceforth referred as the ‘Fast-Food Guideline’) in 2008, which was voluntarily adopted by the food and advertising industries. This guideline restricts fast-food advertising during children’s programmes (such as cartoons) when ≥ 4% of the television audience comprises children aged 4–9 years old [29]. This was followed by a second self-regulatory policy known as the ‘Responsible Advertising to Children Initiative’ (henceforth referred as the ‘Malaysia Pledge’), which was officially launched in 2013 by the Federation of Malaysian Manufacturers Malaysian Food Manufacturing Group (FMM) [30]. Food companies that signed the pledge agreed to advertise only products adhering to their company’s own self-determined nutrient criteria applied during broadcasting when ≥35% of the media audience was <12 years of age [30]. In the third NPANM 2016–2025, the Malaysian government proposed the implementation of ‘hard policies’ to ban television advertisements of unhealthy food to children by 2020 [31] but even up to December 2024, no new regulations have materialized.

Experts in Malaysia raised concerns about the Fast-Food Guideline and Malaysian Pledge, including the use of permissive nutrient criteria and poor compliance by companies [32,33]. The Malaysian government has also acknowledged challenges in implementing the Malaysia Pledge, citing unreliable self-monitoring by industries and a lack of standardized nutrient criteria [34]. Nevertheless, there appears to be no commitment from the public or private sector to strengthen food marketing policies [35,36]. Suggested reasons for this policy inertia include insufficient evidence on the need for mandatory policies and a lack of political will [33].

Globally, the evaluation parameters for television food marketing and benchmarking standards for determining the nutritional quality of advertised products have changed with the introduction of the International Network for Food and Obesity/Non-Communicable Disease Research, Monitoring and Action Support (INFORMAS) food promotion module’s television protocol [37] and the Nutrient Profile Model (NPM) developed by the World Health Organization (WHO) [38]. This underscores the need for updated data collection to inform policy actions needed for the implementation of mandatory regulations and help defend against legal challenges from industries [26]. Furthermore, the World Health Organization (WHO) has called for aligning age protection up to 18 years and adopting the regional Nutrient Profile Model (NPM) to determine marketing permissibility of advertised food products [13,26]. Both factors have yet to be explored in previous studies [20,21], highlighting an important research gap to be addressed. A 2020 study undertaken as part of a 9-country collaboration [22] which used the INFORMAS methodology and benchmarked to the regiospecific NPMs of the WHO examined food marketing on children’s popular television channels across nine Asian countries, including Malaysia. However, this study undertaken during the prevailing COVID-19 lockdowns, likely did not reflect free-living status.

Therefore, this study aimed to investigate the effectiveness of the two self-regulatory policies, which are the Fast-Food Guideline [29] and Malaysia Pledge [30] in restricting the unhealthy television food marketing targeting Malaysian children and adolescents across three consecutive years (2020, 2021, and 2022). A harmonized comparative approach using the INFORMAS food promotion module’s television protocol [37] was adopted and we applied the WHO Western Pacific Regional Office (WPRO) NPM [38] for nutrient profiling. This harmonized approach enabled a retrospective analysis of television food marketing between 2012 and 2022 to examine changes in the food marketing landscape across the decade. The study objective aimed to provide a timely and robust evidence on the current trend of television food advertising for policy makers to evaluate and strengthen existing advertising regulations.

## Methods

### Design and sampling

This study utilized a repeated cross-sectional design to examine television food advertising on Malaysian children’s popular television channels. The top three channels were determined from audience viewership data for 2020, and the same channels were sampled each subsequent year. Data from two of these channels were also available from an earlier study in 2012, using an analogous protocol.

### Data generation

Two datasets were obtained through the study, which were as follows: (i) a main dataset comparing advertisements from 2020, 2021, and 2022, and (ii) a retrospective dataset comparing advertisements in 2012 with the 2022 dataset. For the retrospective comparison, the 2012 dataset was obtained from an earlier study conducted in Malaysia [21].

### Study protocol

This study adopted the same INFORMAS protocol for measuring television food marketing utilized for cross-sectional comparisons of nine Asian countries for which this study’s researchers had collaborated [22,37]. Training in the data collection methodology and description of data procedures and analysis have been detailed elsewhere [22].

### Selection of children’s popular television channels

For the main dataset, the top three popular television channels for children and adolescents aged 4–19 years were selected using Nielsen’s 2019 annual Television Audience Measurement (TAM) data [39]. This age range addressed a research gap identified by a previous Malaysian study [21] for which TAM data was limited to children aged 4–14 years. Extending the age range to 4–19 years, allowed inclusion of children and adolescent which aligns with the WHO recommendations to also protect adolescents (defined as 10–19 years by WHO), as they are also vulnerable to the negative influence of unhealthy food marketing [26]. The selected channels were TV2, TV3 and Astro Ceria. For the retrospective comparison between the 2012 and 2022 datasets, only two television channels (TV2 and TV3) of the top three most popular channels were selected, as these two channels were commonly available for both years.

### Recording protocol

The full recording protocol has been described previously [22]. In summary, for the main dataset, repeated recordings were conducted within a 3-month timeframe in each year: September to November in 2020 and 2021 and October to December in 2022. Convenience sampling was used to select four weekdays and four weekend days for each year within normal schooling weeks, excluding any public or school holidays and special events. All recordings were conducted concurrently for the top three selected channels from 6.00 am to 12.00 am (18 hours per day) using a television capture card and Open Broadcaster Software on three laptops. For the 2012 dataset, recordings were conducted from 6.00 am to 10.00 pm (16 hours per day). To enable the retrospective ten-year comparison, only television broadcasting from 6.00 pm to 10.00 pm was included for 2022 and 2012 in the retrospective dataset.

### Defining peak viewing time

The Nielsen TAM was used to identify peak viewing times (PVT) for children according to the definition of Kelly et al. [19], which refers to the top five 1-hour time slots with the highest children’s viewership on average per weekday and weekend day. For the main dataset, in 2020, 2021 and 2022, the PVT for weekdays was from 6.00 pm to 11.00 pm whilst for weekend days, it was from 7.00 pm to 12.00 am. For the retrospective dataset in 2012, the PVT for weekdays was from 2:00 pm to 3:00 pm and 6:00 pm to 10:00 pm, whereas for weekend days, it was from 2:00 pm to 4:00 pm and 7:00 pm to 10:00 pm [19]. In 2022, the PVT for weekdays was from 5:00 to 10:00 pm, whereas the weekend PVT pattern was similar to 2012.

### Coding of television advertisements

All television advertisements were coded if they were aired during commercial breaks between and during a programme. However, product placements and banner advertisements occurring during the programme were excluded. The included advertisements were coded for broadcast television channel, date, day, program name or category, and time slot. The time slot variable was further separated into PVT and non-PVT, as defined above. Television advertisements identified as food advertisements (Food Ads), including food and non-alcoholic beverages, were further coded for the following information: food product name, product description, and parent company. For Food Ads showing multiple food products, up to three of the most prominent food products were coded per advertisement following the earlier protocol [22]. Additionally, fast food restaurant advertisements were coded for the presence of sugar-sweetened or non-sugar-sweetened beverages (SSBs) shown in the advertisement.

### Content analyses and benchmarking

Advertised food items were classified into food categories based on the NPM proposed by the WHO WPRO [38] (WPRO NPM). This NPM includes energy and nutrient threshold for total energy, total fat, saturated fat, total sugars, added sugars, sodium, and non-sugar sweeteners (NSS) across 21 defined food categories to determine whether the food item should be ‘permitted’ or ‘not permitted’ for advertising to children. According to the model, three food categories were systematically classified as not permitted regardless of nutrient level, which were [1] *Chocolate and sugar confectionery, energy bars, and sweet toppings and desserts* [2]; *Cakes, sweet biscuits and pastries, other sweet bakery products, dry mixes for making such*; and [3] *Energy drinks, tea and coffee*. Culinary ingredients such as uncooked rice and cooking oil were excluded from the content analyses, as they were primarily targeted to homemakers and are unlikely to be requested by children [22].

A market survey was conducted to source the nutrient information required as per the WPRO NPM by visiting physical stores or through digital resources such as the Mintel Global New Products Database [40]. If no food labels were available on advertised products, then national food composition databases of Malaysia and Singapore were referenced to determine their nutrient profile [41,42]. In the main dataset, 4.0% (*n* = 139 out of 3455) of food products’ nutrition information were sourced from these databases. For the retrospective dataset, this figure was 3.1% (*n* = 60 out of 1945). Supplementary Table S4 details the specific food categories for which nutrient information was sourced from food composition databases for each year. In addition, food items such dietary supplements and follow-up formulae for children under 36 months were not included in the WPRO NPM, therefore such items were coded as ‘not applicable’. Food Ads that only showed the food company logo or brand without displaying any food products were classified as ‘Brand only’ (see supplementary Table S2 and S3).

Food Ads were further coded for the presence or absence of persuasive marketing techniques, which included power strategies (PS), and premium offers (PO), as defined by the INFORMAS protocol [37] (refer to Table S1). The combined use of both power strategies and premium offers (PS + PO) within the same advertisement was also examined. Data coding was performed by three researchers, all of whom achieved scores of > 90% as per the recommended intercoder reliability testing described in an earlier study [22].

### Ethics requirement

This study was exempted from receiving ethical approval as it did not involve human participants.

### Statistical analysis

Statistical analysis was performed using SPSS version 29.0 (IBM Corp.). The coded advertisements were aggregated as the total number of advertisements broadcasted per one-hour timeslot for each television channel. The following factors for all years of interest were explored for advertisement rate analysis, including (1) Food Ads as per permitted *vs* not permitted food categories according to WPRO NPM criteria, (2) Food Ads during PVT *vs* non-PVT, (3) top three most frequently advertised food categories of not permitted foods (e.g. *milk drinks*), (4) rate of fast-food advertising, and (5) the use of persuasive marketing techniques in not permitted Food Ads. Data were weighted based on weekday and weekend-day. Descriptive analysis was reported as mean ± SD for the following parameters: rates of food advertising, rate of permitted and not permitted Food Ads, and the ratio of permitted to not permitted Food Ads. As outcomes were not normally distributed, non-parametric analyses were performed to examine the differences in the rate of Food Ads for the investigated factors. These included Wilcoxon signed-rank test to explore the rate difference between permitted and not permitted Food Ads and Mann-Whitney U test to assess the differences between PVT and non-PVT. Persuasive marketing techniques used in not permitted Food Ads were compared between PVT and non-PVT periods by using the Mann-Whitney *U* test. As the data were highly dispersed with high zero counts, zero-inflated negative binomial regression (ZINB) analysis was used to examine the trends in advertising rates across the time periods in both the main and retrospective datasets, that is, (i) 2020 *vs* 2021 *vs* 2022 and (ii) 2012 *vs* 2022. The *p*-value threshold for statistical significance was set at *p* < 0.05.

## Results

### Main data set with top three popular children’s channels

#### Advertising trends for the years 2020, 2021 and 2022

Overall, the broadcasted rate of advertisements on the top three popular children’s television channels were not significantly different between 2020, 2021 and 2022 (22.22 ads/h/channel vs 23.46 ads/h/channel vs 23.03 ads/h/channel, *P*_trend_ > 0.05) respectively (Table 1). Similarly, the overall rates of Food Ads did not differ significantly over time (*P*_*trend*_ > 0.05); 4.07 ads/h/channel in 2020, 4.33 ads/h/channel in 2021 and 4.07 ads/h/channel in 2022. With particular reference to not permitted Food Ads, their rates were also not significantly different between years (2.40, 2.74, and 2.71 ads/h/channel in 2020, 2021 and 2022, respectively; *P*_trend_ > 0.05). In all 3 years, advertising rates for not permitted foods were significantly higher than those for permitted foods (all *p* < 0.001). Permitted food advertising in 2020 as indicated by the ratio of one permitted to every 240 not permitted Food Ads was exceptionally low as in subsequent years, this ratio increased to 1:39 in 2021 and even further to 1:10 in 2022.

**Table 1.** Rate of television advertisements for 2020, 2021 and 2022.

	Advertisement Rates Ads/h/Channel (mean ±SD)		
	Year		
	2020	2021	2022
Total Advertisements	22.22 ± 13.71	23.46 ± 14.05	23.03 ± 13.22
Non-Food Ads^†^	18.14 ± 8.78	19.13 ± 9.46	18.95 ± 9.68
Food Ads^*†^	4.07 ± 6.48	4.33 ± 6.38	4.07 ± 6.73
Permitted (P)^†^	0.01 ± 0.1^a^	0.07 ± 0.30^b^	0.27 ± 0.67^c^
Not Permitted (NP)^†^	2.40 ± 3.88^a^	2.74 ± 3.93^b^	2.71 ± 4.31^c^
P:NP	1: 240	1: 39.1	1: 10.0

Abbreviation: Food Ads = Food Advertisements.

*Food Ads include both solid food and non-alcoholic beverages advertised, nutritional supplements, culinary ingredients, baby food, and follow-up formula for children < 36 months. Advertisements for food companies, retailers, and outlets, even if they do not promote food products, are also included.

^†^Differences in rates for Total Advertisements, Non-Food Ads, Food Ads, Not Permitted Food Ads and Permitted Food Ads between years are not statistically significant (*p* > 0.05).

^a,b,c^Matching superscripts denote significant differences in rates between Permitted Food Ads and Not Permitted Food Ads (*p* < 0.001).

#### Food Ads during peak viewing times in 2020, 2021 and 2022

Rate of not permitted Food Ads were significantly higher during PVT compared to non-PVT for the 3 years (*p* < 0.001) (1.84 times higher in 2020, 1.65 times in 2021 and 1.72 times in 2022) (Table 2). The ZINB regression analysis indicated no significant differences in the trends of the rates of permitted and not permitted Food Ads during both PVT and non-PVT across the 3 years (*P*_*trend*_ > 0.05).

**Table 2.** Comparison of permitted and not permitted food advertising rates during PVT and non-PVT in 2020, 2021, and 2022.

	Food Ads Rates Ads/h/Channel (mean ±SD)			
	Permitted		Not Permitted	
	PVT^†^	Non-PVT^†^	PVT^†^	Non-PVT^†^
2020	0.02 ± 0.15^a^	0.01 ± 0.10^a^	3.58 ± 4.79^b^	1.95 ± 3.37^b^
2021	0.10 ± 0.39	0.05 ± 0.25	3.82 ± 4.68^c^	2.32 ± 3.51^c^
2022	0.50 ± 0.96^d^	0.18 ± 0.50^d^	3.89 ± 5.34^e^	2.26 ± 3.75^e^

Abbreviation: Food Ads = Food Advertisements.

^†^Differences in rates for Permitted (PVT), Permitted (Non-PVT), Not Permitted (PVT) and Not Permitted (Non-PVT) between years are not statistically significant (*p* > 0.05).

^a^Matching superscript denotes significant differences in rates between PVT and non-PVT (*p* < 0.05).

^b,c,d,e^Matching superscript denotes significant differences in rates between PVT and non-PVT (*p* < 0.001).

#### Frequently advertised food categories of not permitted Food Ads in 2020, 2021, and 2022

**Figure 1.**
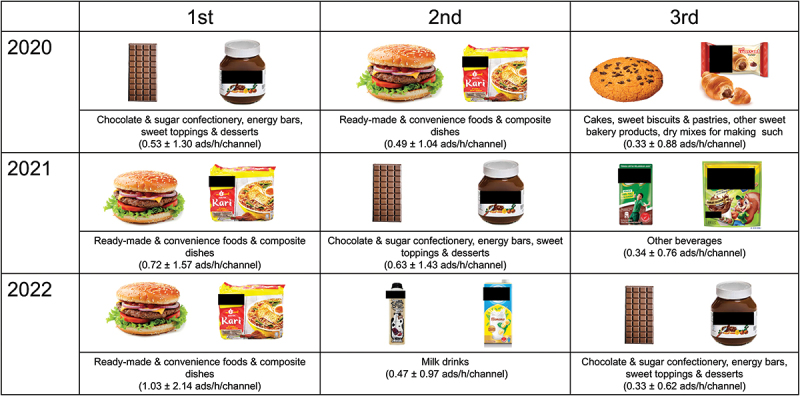
Top three food categories of not permitted foods as per WPRO NPM criteria for the channels TV2, TV3 and Astro Ceria during 2020, 2021 and 2022.

Abbreviation: WPRO NPM = World Health Organization Nutrient Profile Model for the Western Pacific Region

Figure 1 indicates the ranking of the most advertised food categories of not permitted foods for the 3 years. In 2020, *Chocolate & sugar confectionery, energy bars, sweet toppings & desserts* was the most advertised, with an advertisement rate of 0.53 ads/h/channel whereas *Ready-made & convenience foods & composite dishes* became the most frequently advertised category in 2021 and 2022 (0.72 and 1.03 ads/h/channel, respectively). These two food categories were consistently ranked among the top three most advertised foods that were not permitted for all the three years. Other categories in the top three rankings included *Cakes, sweet biscuits & pastries, other sweet bakery products, dry mixes for making such* in 2020 (0.33 ads/h/channel, third rank), *Other beverages* such as chocolate malted drinks in 2021 (0.34 ads/h/channel, third rank), and *Milk drinks* such as flavoured milk in 2022 (0.47 ads/h/channel, second rank).

#### Fast-food advertising in 2020, 2021, and 2022

Advertisements featuring fast-food were broadcasted at a rate of 0.47 ± 1.03 ads/h/channel in 2020, 0.67 ± 1.49 ads/h/channel in 2021, and 1.01 ± 2.11 ads/h/channel in 2022. It was noted that 28% of fast-food advertising included SSBs in 2020 (0.13 out of 0.47 ads/h/channel), 7% in 2021 (0.05 out of 0.67 ads/h/channel), and 63% in 2022 (0.64 out of 1.01 ads/h/channel). All fast-food advertisements were classified as not permitted for the 3 years.

#### Persuasive marketing techniques in not permitted Food Ads in 2020, 2021 and 2022

Persuasive marketing techniques in not permitted Food Ads were evaluated as power strategies and premium offers (Table 3). The use of power strategies was significantly higher during PVT than during non-PVT for all 3 years (*p* < 0.001), with the highest rates observed in 2021 at 2.17 ads/h/channel >1.88 ads/h/channel in 2020 > 1.73 ads/h/channel in 2022. However, the differences in rates across years were not statistically significant (*P*_*trend*_ > 0.05) based on the ZINB regression analysis during both PVT and non-PVT.

**Table 3.** Persuasive marketing techniques use among not permitted Food Ads in 2020, 2021, and 2022.

	Not Permitted Food Ads Rates Ads/h/Channel (mean ±SD)					
	Power Strategies (PS)		Premium Offer (PO)		PS + PO	
	PVT^†^	Non-PVT^†^	PVT^§^	Non-PVT^§^	PVT^†^	Non-PVT^†^
2020	1.88 ± 2.87^a^	0.82 ± 1.49 ^a^	0.77 ± 1.14^d^	0.36 ± 0.78^d^	0.43 ± 0.92^g^	0.15 ± 0.42^g^
2021	2.17 ± 2.69^b^	1.30 ± 1.93^b^	0.73 ± 1.13^e^	0.40 ± 0.85^e^	0.37 ± 0.71^h^	0.23 ± 0.61^h^
2022	1.73 ± 2.25^c^	1.21 ± 1.89^c^	1.05 ± 1.78^f^	0.48 ± 1.12^f^	0.11 ± 0.38^i^	0.06 ± 0.25^i^

Abbreviation: Food Ads = Food Advertisements.

^†^Differences in rates for Power Strategies (PVT), Power Strategies (Non-PVT), PS + PO (PVT) and PS + PO (Non-PVT) between years are not statistically significant (*p* > 0.05).

^§^Differences in rates for Premium Offer (PVT), and Premium Offer (Non-PVT) between years 2020 vs 2022 are statistically significant (*p* < 0.05). However, no significant difference was observed for comparison involving the other years.

^a,b,c,d,e,f,g,h^Matching superscript denotes significant differences in rates between PVT and non-PVT (*p* < 0.001).

^i^Matching superscript denotes significant differences in rates between PVT and non-PVT (*p* < 0.05).

The rates of premium offers in not permitted Food Ads were also significantly higher (*p* < 0.001) during PVT compared to non-PVT for all 3 years (Table 3). As per ZINB regression analysis, comparisons between years indicated premium offers during PVT were only significantly higher in 2022 compared to 2020 (1.05 ads/h/channel vs 0.77 ads/h/channel, *p* = 0.041).

The combined use of both power strategies and premium offers within a single Food Ad was significantly higher during PVT compared to non-PVT. Based on the ZINB regression analysis, the rate of combined usage of both strategies were not significantly different across the 3 years for both children’s PVT and non-PVT periods (*P*_*trend*_ > 0.05).

## Retrospective comparison of food advertising between 2012 and 2022 of two comparable channels

### Rates of Food Ads

The 2022 and 2012 datasets for comparable television channels (TV2 and TV3) revealed advertisements for not permitted foods compared to permitted foods significantly dominated advertising for both years (*p* < 0.001) (Table 4). As per ZINB regression analysis, the rate of not permitted Food Ads was significantly higher in 2012 compared to 2022 (3.78 vs 3.34 ads/h/channel, *p* < 0.001). The rate of permitted Food Ads remained low for both 2012 and 2022 (*p* = 0.139).

**Table 4.** Rate of permitted and not permitted food advertising in 2012 and 2022.

	Food Ads Rates Ads/h/Channel (mean ±SD)		
	WPRO		
	Permitted (P)^†^	Not Permitted (NP) ^§^	P: NP
2012	0.29 ± 0.57^a^	3.78 ± 3.77^a^	1: 13.0
2022	0.39 ± 0.79^b^	3.34 ± 4.91^b^	1: 8.6

Abbreviation: Food Ads = Food Advertisements.

^†^Differences in rates for Permitted Food Ads between years are not statistically significant (*p* > 0.05).

^§^Differences in rates for Not Permitted Food Ads between years 2012 and 2022 are statistically significant (*p* < 0.001).

^a,b^Matching superscripts denote significant differences in rates between Permitted Food Ads and Not Permitted Food Ads (*p* < 0.001).

### Food Ads during peak viewing times

In 2022, the rate of advertising for not permitted foods was significantly higher during PVT compared to non-PVT (4.63 vs 2.75 ads/h/channel, *p* < 0.001). However, in 2012 advertising rates for not permitted foods were not significantly different between both PVT and non-PVT (3.73 vs 3.80 ads/h/channel, *p* = 0.228) (Table 5). Based on the ZINB regression analysis, the rates of not permitted Food Ads during PVT were significantly higher in 2022 compared to 2012 (4.63 vs 3.73 ads/h/channel, *p* < 0.001).

**Table 5.** Retrospective comparison of permitted and not permitted food advertising rates during PVT and non-PVT.

	Food Ads Rates Ads/h/Channel (mean ±SD)			
	Permitted		Not Permitted	
	PVT^†^	Non-PVT^†^	PVT^§^	Non-PVT^§^
2012	0.39 ± 0.55^a^	0.25 ± 0.57^a^	3.73 ± 3.16	3.80 ± 4.02
2022	0.69 ± 1.07^b^	0.25 ± 0.58^b^	4.63 ± 5.65^c^	2.75 ± 4.44^c^

Abbreviation: Food Ads = Food Advertisements.

^†^Differences in rates for Permitted (PVT), and Permitted (Non-PVT) between years are not statistically significant (*p* > 0.05).

^§^Differences in rates for Not Permitted (PVT), and Not Permitted (Non-PVT) between years 2012 and 2022 are statistically significant (*p* < 0.001).

^a,b,c^Matching superscript denotes significant differences in rates between PVT and non-PVT (*p* < 0.001).

### Popular Food categories of not permitted Food Ads

**Figure 2.**
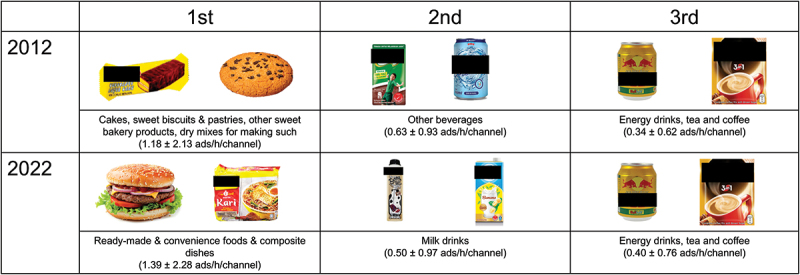
Top three food categories of not permitted foods as per WPRO NPM criteria for the channels TV2 and TV3 during 2012 and 2022.

Abbreviation: WPRO NPM = World Health Organization Nutrient Profile Model for the Western Pacific Region

In 2012, the most frequently advertised food categories of not permitted foods were *Cakes, sweet biscuits & pastries, other sweet bakery products, dry mixes for making such* (1.18 ads/h/channel), *Other beverages* (0.63 ad/h/channel), and *Energy drinks, tea and coffee* (0.34 ads/h/channel) (Figure 2). By 2022, the ranking had shifted to *Ready-made & convenience foods & composite dishes* (1.39 ads/h/channel), *Milk drinks* (0.50 ads/h/channel), and *Energy drinks, tea and coffee* (0.40 ads/h/channel).

### Fast-food advertising in 2012 vs 2022

Advertisements featuring fast-food were broadcasted at a rate of 0.21 ± 0.47 ads/h/channel in 2012 compared to 1.33 ± 2.23 ads/h/channel in 2022. In 2012, 57% of fast-food advertisements (0.12 out of 0.21 ads/h/channel) included SSBs, and this increased to 64% in 2022 (0.85 out of 1.33 ads/h/channel). All fast-food advertisements were classified as not permitted.

### Persuasive marketing techniques in not permitted Food Ads

In 2022, the use of power strategies and premium offers in not permitted Food Ads was significantly higher during PVT compared to non-PVT (*p* < 0.001) (Table 6). Whereas in 2012, rate differences for these marketing techniques used in PVT and non-PVT were not significant (*p* > 0.05). According to ZINB regression analysis, the rates of power strategies during PVT were significantly higher in 2022 compared to 2012 (2.06 vs 1.88 ads/h/channel, *p* = 0.002).

**Table 6.** Persuasive marketing techniques use among not permitted Food Ads in 2012 and 2022.

	Not Permitted Food Ads Rates Ads/h/Channel (mean ±SD)					
	Power Strategies (PS)		Premium Offer (PO)		PS + PO	
	PVT^§^	Non-PVT^§^	PVT^†^	Non-PVT^§^	PVT^†^	Non-PVT^†^
2012	1.88 ± 1.57	2.32 ± 2.90	0.57 ± 0.87	0.64 ± 1·08	0.40 ± 0.71	0.58 ± 1.06
2022	2.06 ± 2.55^a^	1.27 ± 2.21^a^	1.20 ± 1.76^b^	0.70 ± 1·30^b^	0.13 ± 0.39	0.10 ± 0.33

Abbreviation: Food Ads = Food Advertisements.

^†^Differences in rates for Premium Offer (PVT), PS + PO (PVT) and PS + PO (Non-PVT) between years are not statistically significant (*p* > 0.05).

^§^Differences in rates for Power Strategies (PVT), Power Strategies (Non-PVT), and Premium Offer (Non-PVT) between years 2020 and 2022 are statistically significant (*p* < 0.05).

^a,b^Matching superscript denotes significant differences in rates between PVT and non-PVT (*p* < 0.001).

## Discussion

Despite the rise of digital media in recent years, television remains the most accessible medium for children’s media consumption in Malaysia with over 99% of households having access to television [43,44]. We examined for the first time in Malaysia, a 3-year sequential scenario of food advertising trends on children’s popular television channels by utilizing a robust and standardized monitoring protocol benchmarked to a WHO NPM to categorize the healthfulness of these advertised food products [37,38] Using the same protocol, we also performed a retrospective comparison of such television food advertising, bridging a decade between 2022 and earlier data from 2012, using comparable television channels and recording hours. Valuable insights were generated by these two datasets.

Primarily, we found that unhealthy Food Ads continued to dominate the television food advertising scene for all the years measured, despite the presence of two self-regulatory policies [29,30]. Further, advertisers in 2022 were concentrating unhealthy Food Ads during broadcasting time with the highest children’s audience (i.e. PVT) compared to a decade earlier in tandem with persuasive marketing techniques dominating this time segment. Specifically, the main dataset included a period of protracted lockdown and school closures during the COVID-19 pandemic of 2020 [45] with the subsequent 2 years (i.e. 2021 and 2022) of normalized free-living status. Television marketing pattern did not differ between the lockdown year of 2020 and the subsequent years, 2021 and 2022. Moreover, for these years the most featured unhealthy advertising was of fast-food restaurants with the rate more than doubling from 2020 to 2022 (from 0.47 Ads/h/Channel to 1.01 Ads/h/Channel). These rates are comparable to other Asian countries ranging from Indonesia (0.3 Ads/h/Channel) to China (1.0 Ads/h/Channel) [18]. However, data interpretation is limited by methodological differences, such as difference in number of recording days and recording hours.

Specific to the retrospective dataset, a decline in the overall rate of not permitted Food Ads was observed from 2012 to 2022 (3.78 ± 3.77 vs 3.34 ± 4.91 Ads/h/Channel, *p* < 0.001) but this may be attributed to the emergence of ‘Brand only’ marketing in 2022 broadcasted at a rate of 0.36 ± 0.69 Ads/h/Channel (Table S3). ‘Brand only’ Food Ads featuring only company logos without displaying actual food products were absent in 2012. This form of marketing has also been observed in another Asian country, the Philippines, where such marketing accounted for 0.4% of all television Food Ads [46]. Fast-food advertising increased six-fold, as indicated by the retrospective comparison between 2012 and 2022 datasets (0.21 ads/h/channel *vs* 1.33 ads/h/channel). A notable feature of this marketing was the combined strategy of promoting SSBs within fast-food advertisements (57% in 2012 *vs* 64% in 2022).

Broadcasting hours with the highest children’s audience designated as PVT is prime time for maximal potential exposure to marketing activities [47]. In the main dataset, we found the rate of not permitted Food Ads nearly doubled during children’s PVT compared to non-PVT which is in line with other studies observing the concentration of unhealthy Food Ads during PVT [19,46]. Additionally, there was a greater use of power strategies and premium offers during PVT. This phenomenon was particularly evident for the 2022 dataset compared to 2012 which showed no significant difference between PVT and non-PVT period. Using persuasive marketing techniques in unhealthy food marketing during PVT is exploitative in nature, as children have a relatively limited cognitive ability to recognize the selling and persuasive intent of marketing compared to adults [48,49]. These marketing techniques have been shown to be effective in influencing children’s food choices, attitudes, and ultimately increasing their consumption of unhealthy foods [49,50]. Of significance, this PVT period typically occurred after 6 pm in Malaysia as schooling, tuition and religious classes commonly occupy the earlier hours of children.

*Has the introduction of two self-regulatory policies, namely the Fast-Food Guideline in 2008 and the Malaysia Pledge in 2013 been effective in limiting unhealthy food advertising and usage of persuasive marketing techniques on television in Malaysia?* The Pledge was enacted in 2013 as a call to action by the food industry after earlier studies in Malaysia raised concerns on unhealthy food marketing targeting children’s television [20]. Although the FMM has claimed a high compliance rate of 98% of food companies signatory to the Malaysia Pledge [51], our study disclosed the current television food marketing scenario in Malaysia remains saturated with unhealthy food advertising. This Malaysian scenario aligns with other Asian countries such as Thailand [52], the Philippines [46], and India [53] commonly reporting unhealthy foods dominating television advertising compared to healthy foods despite implementing similar responsible marketing policy introduced by the International Food & Beverage Alliance [54]. A policy review in Malaysia has revealed a lack of interest of companies mostly selling unhealthy food to voluntarily participate in the Malaysia Pledge [33]. Additionally, as the Pledge is not legislated, companies do not face legal punishment for violations, and the lack of monitoring in Malaysia further exacerbates this situation, ultimately raising questions about the credibility and effectiveness of the Pledge [33].

Furthermore, the fast-food advertising rate has increased in the current television marketing scenario despite the introduction of the government-led Fast-Food Guideline [29] in 2008, indicating another failure of regulation. The overall increase in fast-food advertising as indicated by our study is deeply concerning, as fast-food consumption among children is likely to lead to excess weight gain [55] with the additional marketing of SSBs embedded in fast-food advertisements further exacerbating the issue of excess calorie consumption [56]. Worryingly, a national survey in Malaysia indicated 82.8% of adolescents consumed fast foods at least once per week [57]. Additionally, the voluntary nature of the Fast-Food Guideline, and the lack of government led monitoring further undermines its effectiveness [33].

Overall, our study highlights the inadequacy of the current self-regulatory policies in restricting the unhealthy food marketing toward children in television, especially during PVT. Self-regulatory policies have been shown to be ineffective in restricting unhealthy food marketing to children on television, with mandatory policies being more likely to be effective [58–60]. Local legal experts have also called for a mandatory approach to restrict unhealthy food marketing as a legitimate intervention to combat obesity in Malaysia [61]. Both the Malaysia Pledge and the Fast-Food Guideline fall short of aligning with the age protection recommendations for children up to 18 years old, as advocated by the WHO [13,26]. In context, the Malaysia Pledge applies a narrow criterion of only restricting unhealthy food advertising during broadcasting with children under 12 years old constituting ≥ 35% of the total audience [30]. Further, the Fast-Food Guideline only applies to children aged 4–9 years old and targets children’s television programmes with ≥ 4% of viewers in this age group [29]. Adolescents are equally vulnerable to the effects of unhealthy food marketing due to their unique social and developmental characteristics, and they are at a higher risk due to their increased purchasing power compared to younger children [34,62]. Incidentally, it should also be pointed out that these policies referenced permissive nutrient criteria [32,33] that did not comply to the WHO recommendations [13].

Countries that have adopted mandatory approaches to food marketing regulations have shown promising results. For example, South Korea’s mandatory regulation, known as the Special Act on Safety Management of Children’s Dietary Life, restricts unhealthy food advertising on television during children’s peak viewing period from 5:00 pm to 7:00 pm [26,63]. This regulation has prompted food companies to reformulate their products to make them healthier, thereby improving their food environment [63]. Additionally, Chile saw a significant decrease in the prevalence of unhealthy television advertisements after implementation of a mandatory law that bans the marketing of high energy, saturated fats, sugar, or sodium foods to children younger than 14 years in the media [64].

The strength of this study includes the use of a robust and standardized monitoring protocol of INFORMAS and incorporating the NPM for the regiospecific WPRO of the WHO. This approach aligns with the recent call to member states to adopt the WHO regional NPM to restrict unhealthy foods marketed to children [13,26]. The adoption of a standardized monitoring protocol across multiple time points enabled data comparisons across different years, including the retrospective analysis before and after a decade of implementing the Malaysia Pledge. This study’s major outcome should be used to advocate for mandatory regulation in Malaysia, including aligning age protection up to 18 years old and adoption of a standardized NPM benchmark such as the WPRO NPM [38].

Limitations must be recognized with our use of the 2012 dataset in the retrospective analysis, which was collected prior to the development of the current INFORMAS protocol and WPRO NPM, resulting in some methodological differences between the 2012 and 2022 datasets. First, television channels were sampled in 2012 using the viewership data for children aged 4–14 years as opposed to the 4–19 years old criteria set in 2022. Second, the television broadcast in 2012 were recorded for 16 hours per day (6:00 am to 10:00 pm) compared to the 18 hours (6:00 am to 12:00 am) in 2022. These limitations were minimized by selecting two television channels, TV2 and TV3, consistent to being among the top three most popular channels for both 2012 and 2022 to facilitate the retrospective analysis. Additionally, the broadcasting hours for the retrospective analysis were limited to only 16 hours (6:00 am to 10:00 pm) to match the recording availability in the 2012 dataset. Finally, the 2012 dataset was coded using the current protocol and the WPRO NPM to ensure comparability with the 2022 dataset.

## Conclusion

This study provides compelling evidence that despite the implementation of two self-regulatory policies, unhealthy food advertising continues to dominate children’s popular television channels, with healthy Food Ads being nearly non-existent. Advertisers have increasingly shifted toward concentrating their advertising during children’s peak viewing time. The Fast-Food Guideline has also proven ineffective as fast-food consistently ranked among the top three most advertised unhealthy food categories in the main dataset. Altogether, our findings provide strong evidence in support of the implementation of government-led mandatory regulations that protect children from exposure to unhealthy food advertising on television particularly as current self-regulatory policies have failed to do so.

## Supplementary Material

STROBE_checklist_Malaysia TV Paper.docx

GHA Supplementary File V2.docx

## Data Availability

Data is available upon reasonable request from the corresponding author.
