# Supplementary material for: Scrutinizing the impact of two self-regulation policies on unhealthy food marketing in children’s popular television in Malaysia: a multiple-year repeated evaluation using a harmonized protocol
Source: Glob Health Action. 2025 Aug 21;18(1):2543617. doi: 10.1080/16549716.2025.2543617 (PMC12372512; doi:10.1080/16549716.2025.2543617)
Supplement: GHA Supplementary File V2.docx [file ZGHA_A_2543617_SM8410.docx]

**Table S1: Power strategies and premium offers as per the INFORMAS protocol**

| **Power Strategies** | **Premium Offer** |
| --- | --- |
| Cartoon/Company owned character e.g. M&Ms | Game and App downloads |
| Licensed character e.g. Dora the explorer | Contests |
| Amateur sportsperson e.g. person playing sport | Pay 2 take 3 or other |
| Celebrity (non-sports) e.g. Jamie Oliver | 20% extra or other |
| Movie tie-in e.g. Shrek | Limited edition |
| Famous sportsperson/team e.g. All Blacks | Social charity |
| Non-sports/historical events/festivals e.g. Christmas, Anzac Day | Gift or collectable |
| ‘For kids’ e.g. image of a child engaging with the product/ consuming the product, ‘great for school lunches’, ‘for school lunchboxes’ | Price discount |
| Awards e.g. Best Food Award 2014, award winning, number one best-selling’ | Loyalty programs |
| Sports event |  |

**Table S2: Rate of food advertisements as per “Not Applicable”, “Insufficient NIP” and “Brand Only” categories for the main dataset.**

|  | **Advertisement Rates**  **Ads/h/Channel (mean** ±**SD)** | | |
| --- | --- | --- | --- |
|  | Year | | |
|  | 2020 | 2021 | 2022 |
| Not Applicable | 0.94 ± 2.16 | 0.83 ± 1.90 | 0.78 ± 1.94 |
| Insufficient NIP | 0.13 ± 0.41 | 0 | 0.07 ± 0.27 |
| Brand Only | 0.59 ± 1.25 | 0.70 ± 1.45 | 0.24 ± 0.60 |

Abbreviation: NIP= Nutrition Information Panel

Note: “Not Applicable” are food product that could not be benchmarked to the WHO NPM such as dietary supplements; “Insufficient NIP” is a product without key nutrition information as required by the nutrient thresholds of the WHO NPM; “Brand Only” are advertisements showing only the food company’s brand without showing the food product.

**Table S3: Rate of food advertisements as per “Not Applicable”, “Insufficient NIP” and “Brand Only” categories for the retrospective dataset.**

|  | **Advertisement Rates**  **Ads/h/Channel (mean** ±**SD)** | |
| --- | --- | --- |
|  | Year | |
|  | 2012 | 2022 |
| Not Applicable | 0.56 ± 0.93 | 1.15 ± 2.33 |
| Insufficient NIP | 0.20 ± 0.44 | 0.10 ± 0.33 |
| Brand Only | 0 | 0.36 ± 0.69 |

Abbreviation: NIP= Nutrition Information Panel

Note: “Not Applicable” are food product that could not be benchmarked to the WHO NPM such as dietary supplements; “Insufficient NIP” is a product without key nutrition information as required by the nutrient thresholds of the WHO NPM; “Brand Only” are advertisements showing only the food company’s brand without showing the food product.

**Table S4: Occurrence of food products without nutrition labels within food categories for the recorded years.**

| **Year** | **Food Categories** | ***n^*^* (%)** |
| --- | --- | --- |
| ***Main Dataset*** | | 139 (100) |
| 2020 | Savoury snacks | 11 (7.9) |
| 2021 | Ready-made and convenience foods and composite dishes | 71 (51.1) |
| 2022 | Ready-made and convenience foods and composite dishes | 57 (41.0) |
| ***Retrospective Dataset*** | | 60 (100) |
| 2012 | Ready-made and convenience foods and composite dishes | 14 (23.3) |
| 2022 | Ready-made and convenience foods and composite dishes | 46 (76.7) |

*These items lacked nutrition labels and nutrition information were referenced from national food composition databases of Malaysia (Malaysia Nutrition Division, n.d.) and Singapore (Singapore Health Promotion Board, 2024).

**References**

…1 Malaysia Nutrition Division. Malaysia Food Composition Database. n.d. [cited 3 July 2024]. Available from: https://myfcd.moh.gov.my/index.html

…2 Singapore Health Promotion Board. Energy & Nutrient Composition of Food. 2024 [cited 3 September 2024]. Available from: https://focos.hpb.gov.sg/eservices/ENCF/
